# Where to Buy "The Hospital"

**Published:** 1887-09-17

**Authors:** 


### WHERE TO BUY "THE HOSPITAL."

SEVERAL correspondents have complained that they cannot get a copy of THE HOSPITAL from the newsagents or booksellers. Nurse M. D. Walker puts it in this way:

"I should like to draw your attention to the fact of how very difficult it is for private nurses to get THE HOSPITAL. I have never yet been able to get a copy at any stationer's without ordering it previously, which is almost an impossibility, as I seldom know from week to week where I may be, and have often been to as many as six shops on the chance of getting it, without avail. Many newsvendors don't even know of it, and others say that they only get them to order, as there is some difficulty in getting the unsold copies allowed for. The difficulty is not confined to one quarter: it is the same everywhere, for I have asked for a copy at Charing Cross Station ere now at twelve o'clock on Friday, and they had none left; so even Smith's bookstalls cannot be well supplied. At South Kensington Station last week the boy said they did not keep it there. As far as I am concerned I would have it straight from you had I a settled address; but I think you will see how very awkward it is for a private nurse to make an arrangement of the kind, and I am sure you will do your best to meet the want, although I quite see how difficult it must be with a demand so irregular. But in my little experience many nurses have given up taking it, because it is such a bother to get, they say. It is often impossible for a private nurse either to get it, or send for it on the Friday, and it may be some days after before she is able to do so. I for one look forward to seeing it as soon as I can get it, being much interested in the progress of the Pension Fund, also the general information contained therein."

*** We publish the above as a sample of very many letters sent to us complaining of the difficulty of procuring THE HOSPITAL. In order, as far as possible, to obviate such difficulty in future, we give this week a list of firms who supply the paper regularly. The list will be found on page v of the advertisements. Anyone failing to get a copy should write to the publisher, stating the facts.

